# Data on fossil fuel availability for Shared Socioeconomic Pathways

**DOI:** 10.1016/j.dib.2016.11.043

**Published:** 2016-11-18

**Authors:** Nico Bauer, Jérôme Hilaire, Robert J. Brecha, Jae Edmonds, Kejun Jiang, Elmar Kriegler, Hans-Holger Rogner, Fabio Sferra

**Affiliations:** a Potsdam Institute for CIimate Impact Research (PIK), Germany; b Mercator Research Institute on Global Commons and Climate Change (MCC), Germany; c University of Dayton, Department of Physics, Ohio, USA; d Pacific National Northwestern Laboratory (PNNL), Maryland, USA; e Energy Research Institute (ERI), Beijing; f International Institute for Applied System Analysis (IIASA), Austria; g Centro Mediterraneo dei Cambiamenti Climatici (CMCC), Italy; h Climate Analytics, Germany

**Keywords:** Energy economics, Shared Socio-economic Pathways (SSPs), Fossil fuel sector, Coal, Oil, Gas, Integrated Assessment Models, Extraction cost

## Abstract

The data files contain the assumptions and results for the construction of cumulative availability curves for coal, oil and gas for the five Shared Socioeconomic Pathways. The files include the maximum availability (also known as cumulative extraction cost curves) and the assumptions that are applied to construct the SSPs. The data is differentiated into twenty regions. The resulting cumulative availability curves are plotted and the aggregate data as well as cumulative availability curves are compared across SSPs. The methodology, the data sources and the assumptions are documented in a related article (N. Bauer, J. Hilaire, R.J. Brecha, J. Edmonds, K. Jiang, E. Kriegler, H.-H. Rogner, F. Sferra, 2016) [1] under DOI: http://dx.doi.org/10.1016/j.energy.2016.05.088.

**Specifications Table**

**Table:** 

Subject area	*Economics*
More specific subject area	Energy economics, Shared socio-economic pathways (SSPs), fossil fuel sector, coal, oil, gas, Integrated Assessment Models, extraction cost
Type of data	*Data files in EXCEL format.*
How data was acquired	*Data bases, literature review*
Data format	*Raw data, additional assumptions and resulting data*
Experimental factors	*Not applicable.*
Experimental features	*Not applicable.*
Data source location	*Not applicable.*
Data accessibility	*Data is within this article*

**Value of the data**

• Cumulative extraction cost curves can be used in models.
• Alternative assumptions can be applied to the data to formulate alternative interpretations of SSPs.
• Data files can be updated as improved data becomes available.
• Data can be used for comparing assumptions across models.

## 1 Data

Files contain original data, assumptions for formulating the alternative scenarios and the resulting cumulative availability curves for coal, oil and gas. The spreadsheet names are clearly identifying the different pieces of information. At the beginning there is also guidance on how to read the data.

## 2 Experimental design, materials and methods

The data is a collection and harmonization of fossil fuel and resources data as well as costs reported in the literature. The basis is the Global Energy Assessment data set that is up-dated according to changes that are fully documented in the article. The assumptions that are applied on the data to derive SSP specific cumulative availability curves are fully included and the article also reports the reasons for the various assumptions. Finally, all resulting cumulative availability curves are fully included giving the data and providing figures.

## References

[1] Bauer N., Hilaire J., Brecha R.J., Edmonds J., Jiang K., Kriegler E., Rogner H.-H., Sferra F. (2016). Assessing global fossil fuel availability in a scenario framework. Energy.

