# Supplementary material for: Data on fossil fuel availability for Shared Socioeconomic Pathways
Source: Data Brief. 2016 Nov 18;10:44–6. doi: 10.1016/j.dib.2016.11.043 (PMC5137328; doi:10.1016/j.dib.2016.11.043)
Supplement: Supplementary file 1 — Supplementary material [file mmc1.zip › Edmonds - Conflict of Interest Signed Ltr.pdf]

Pacific Northwest NATIONAL LABORATORY
Proudly Operated by Battelle Since 1965
UNIVERSITY OF MARYLAND

JOINT GLOBAL CHANGE RESEARCH INSTITUTE
5825 University Research Court, Suite 3500
College Park, MD 20740-3823
(301) 314-6737
www.globalchange.umd.edu

June 17, 2016

Letter for Nico Bauer on Conflict of Interest

I wish to confirm that I have no known conflicts of interest associated with this publication and there has been no significant financial support for this work that could have influenced its outcome.

I confirm that I have read and approved the manuscript and that I know of no other person who satisfied the criteria for authorship but is not listed. I further confirm that the order of authors listed in the manuscript has been approved by all of us.

I confirm that I have given due consideration to the protection of intellectual property associated with this work and that there are no impediments to publication, including the timing of publication, with respect to intellectual property. In so doing I confirm that I have followed the regulations of my institution concerning intellectual property.

I understand that the Corresponding Author is the sole contact for the Editorial process (including Editorial Manager and direct communications with the office). He is responsible for communicating with the other authors about progress, submissions of revisions and final approval of proofs. I confirm that I have provided a current, correct email address, jae@pnnl.gov, which is accessible by the Corresponding Author and which has been configured to accept email from Nico Bauer (nicolasb@pik-potsdam.de).

Sincerely,

Jae Edmonds
Battelle Fellow and Chief Scientist
Joint Global Change Research Institute
Pacific Northwest National Laboratory
